# Revealing the Genetic Impact of the Ottoman Occupation on Ethnic Groups of East-Central Europe and on the Roma Population of the Area

**DOI:** 10.3389/fgene.2019.00558

**Published:** 2019-06-13

**Authors:** Zsolt Bánfai, Béla I. Melegh, Katalin Sümegi, Kinga Hadzsiev, Attila Miseta, Miklós Kásler, Béla Melegh

**Affiliations:** ^1^Department of Medical Genetics, Clinical Centre, University of Pécs, Pécs, Hungary; ^2^Szentágothai Research Centre, University of Pécs, Pécs, Hungary; ^3^Department of Laboratory Medicine, Medical School, University of Pécs, Pécs, Hungary; ^4^National Institute of Oncology, Budapest, Hungary

**Keywords:** population genetics, genome-wide data, population structure, ancestry estimation, admixture, East-Central Europe, historical aspects

## Abstract

History of East-Central Europe has been intertwined with the history of Turks in the past. A significant part of this region of Europe has been fallen under Ottoman control during the 150 years of Ottoman occupation in the 16–17th centuries. The presence of the Ottoman Empire affected this area not only culturally but also demographically. The Romani people, the largest ethnic minority of the East-Central European area, share an even more eventful past with Turkish people from the time of their migration throughout Eurasia and they were a notable ethnic group in East-Central Europe in the Ottoman era already. The relationship of Turks with East-Central European ethnic groups and with regional Roma ethnicity was investigated based on genome-wide autosomal single nucleotide polymorphism data. Population structure analysis, ancestry estimation, various formal tests of admixture and DNA segment analyses were carried out in order to shed light to the conclusion of these events on a genome-wide basis. Analyses show that the Ottoman occupation of Europe left detectable impact in the affected East-Central European area and shaped the ancestry of the Romani people as well. We estimate that the investigated European populations have an average identity-by-descent share of 0.61 with Turks, which is notable, compared to other European populations living in West and North Europe far from the affected area, and compared to the share of Sardinians, living isolated from these events. Admixture of Roma and Turks during the Ottoman rule show also high extent.

## Introduction

According to genome-wide studies based on sex chromosomes, Europeans have two main features in their genetic makeup regarding the paternal and maternal lineages. Investigations of Y haplogroups show that there is geographical pattern in the Y haplogroup characteristics of distinct European regional populations. East-Central Europe and the rest of East Europe separate clearly from the Northern and Western parts of Europe regarding some major haplogroups. The R1b haplogroup is primarily a characteristic of West Europeans and its significance strongly decreases toward East Europe, where R1a is the predominant R haplogroup. R1b is Celtic, Saxon, Basque and Frisian related, originating from the Neolithic. R1a is mainly a Slavic derived haplogroup (Balaresque et al., 2010; Cruciani et al., 2011; Myres et al., 2011; Rocca et al., 2012; Underhill et al., 2015). Most of the East-Central European ethnic groups share R1a and R1b to a similar extent (Kayser et al., 2005). Western and mainly Northern groups show high extent of the I1 haplogroup, which is Germanic derived, Eastern and East-Central European populations possess mainly the I2, a Slavic derived haplogroup (Rootsi et al., 2004). E3b and J haplogroups are represented with a significant proportion in the genetic makeup of East-Central Europeans, distinguishing them from other European populations (Semino et al., 2004). Both haplogroups are connected predominantly to the Middle Eastern region. E3b is connected mainly to Middle Eastern and North African ancestries, haplogroup J indicates Arab, Greco-Roman, Anatolian and Mesopotamian ancestries (Cruciani et al., 2004; Semino et al., 2004; Battaglia et al., 2009). Studies based on mitochondrial DNA showed us that the maternal lineage does not reflect any patterns in connection with the geographical location of populations, mtDNA haplogroups are ubiquitous all over Europe with the predominance of haplogroups H, U, and T (Torroni et al., 2000). This could be the result of old socio-cultural factors present in Europe, such as polygyny or even patrilocality. The latter refers to the social system where the married couple resides near the husband’s birthplace. Summarizing, the genetic properties of European populations based on the paternal lineage reflect their geographical location, so East-Central Europe is well-separated from West and North Europe and has also specific features due to Middle Eastern effects, which also makes it possible to separate the region from East Europe.

Roma (Romani, Gypsies) are a traditionally itinerant, diverse in culture and dispersed ethnic group with a population counting at least 10–15 million individuals worldwide, with the largest population size in Europe (Liégeois, 1994). According to estimations, the Roma population in Europe counts approximately 12 million individuals (European Union Agency for Fundamental Rights, 2016). This makes them the largest ethnic minority in Europe by far. The fact that the European Roma reside in large numbers in the East-Central European countries makes them an important ethnicity of the region along with the main ethnic groups of East-Central European countries (Liégeois, 1994).

Europe has a long common history with Turks, which dates back to the dawn of the Ottoman era, when Turks began to conquer the Eastern regions of Europe. The Ottoman Empire was founded by Oghuz Turks led by Osman I in Anatolia in the 13th century (Kermeli, 2010). As a result of their subsequent conquering campaigns, the power of the Ottoman Empire increased gradually on the Anatolian peninsula. Ottomans invaded also their neighboring regions and the first target area of these conquest campaigns was the Balkans. When the Ottoman Emperor Murad I defeated the united Serbian army at Kosovo in 1389, Europe became wide open to the Ottoman expansion into the continent (Elsie, 2004). By the dawn of the 16th century, its territory incorporated a significant part of East-Central Europe with a Northern border at the top of the Pannonian (or Carpathian) basin, territory of The Kingdom of Hungary. About one third of the area of the Kingdom fell to Ottoman control in the 16th and 17th centuries for about 150 years. A significant part of the Kingdom became vassal of the Ottoman Empire as did parts of the adjacent Eastern countries like Ukraine and Moldavia (Gábor, 2013). As we know, Turks stayed in East-Central Europe at least for 5 generations, and approximately 80,000 Muslims settled in the Ottoman controlled region of the Kingdom of Hungary (Sugar, 1977). It was a part of the Ottoman conquest strategy that they colonized the militarily occupied areas in order to establish a solid base for further conquering campaigns and to consolidate their position and power in a newly conquered area (Eminov, 1997). This colonization strategy resulted in some notable Turkish settlements e.g., the Balkan Turk communities. Most of the major Balkan towns, especially, which lay near to transportation routes, became overwhelmingly Muslim in demographic composition until the 18–19th centuries (Eminov, 1997). This relatively large time interval, colonization and settlement processes could allow and initiate significant admixture between the Turkish people and Europeans, which could leave detectable Turkish ancestry in their genetic makeup.

Researchers on the field of history show us that Romani people share an even more eventful history with Turks. Romani people share a common history with Turks not only because they lived in East-Central European countries under Ottoman occupation, but because their migration route included also Oghuz Turk occupying area. After their exodus from Northwest India, Roma reached the Caucasus region, the Middle East, including the Anatolian peninsula, in the 12th and the Balkans in the 13th centuries (Mendizabal et al., 2012). They arrived in the central region of Europe only in the 14th century, fleeing from the Ottoman conquering campaigns. In the 16th century, when a significant part of East-Central Europe was claimed by the Ottoman Empire, the Roma people were a significant ethnic minority of the area already (Crowe, 2007).

The conquering campaigns of the Ottoman Empire played an important role in the history of East-Central Europe. The effect of the Ottoman occupation left its mark on these parts of the European continent not only in a demographic and cultural but supposedly also in a genetic manner. Although, the genetic impact on populations living in the formerly Ottoman occupied area remained unrevealed and has not yet been studied using genome-wide autosomal marker data. Our aim was to investigate the relationship of Turks with the major ethnic groups of East-Central European countries and with the local Roma ethnic minority using genome-wide single nucleotide polymorphism (SNP) array data. We attempted to measure the Turkish ancestry in these populations. However, according to the history of East Europe, other Turkic ancestry can also be derived from several nomadic Turkic tribes, such as the Cumans, Kipchacks, Pechenegs and Bulgars invading and ruling West Eurasia (including parts of East Europe as well), and also the Khazars, of which a group called Khabars joined to the migrating ancestors of Hungarians (Sugar et al., 1990). Therefore, significant Turkic related ancestry of East Europeans could originate from before the time of Ottoman occupation. Ancestors of East European populations could have admixed with such Turkic people, which can affect our investigation (Golden, 2003; Cole, 2011).

In summary, our study concentrates on the effect of the Ottoman Empire on the formerly Ottoman occupied area of East-Central Europe in a genetic level, including major ethnic groups of regional countries and also Romani people, the largest ethnic minority of the area. We attempted to reveal their presumed admixture with Turks.

## Materials and Methods

### Datasets

The populations investigated in this study were obtained from various sources. Our Roma data consists of upon request available data and in international collaboration collected and genotyped data (Mendizabal et al., 2012; Moorjani et al., 2013). Both Roma datasets were genotyped on an Affymetrix 1M chip and consisted of 152 and 27 Roma individuals. Roma individuals with East-Central European origin (Bulgaria, Croatia, Greece, Hungary, Romania, Serbia, and Slovakia) were extracted from the two datasets and merged together. The merged Roma dataset consisted of 158 Roma samples featuring 599,472 SNPs. Hungarian samples, one of the East-Central European populations, were applied from our repository. The dataset consisted of 251 individuals and was genotyped also on the Affymetrix Genome-wide Human SNP Array 6.0 platform. According to the preliminary clustering analyses, Hungarian samples falling off from the Hungarian cluster, and therefore showing significant foreign ancestry, were removed from the data. The thinned Hungarian dataset contained 238 individuals with 898,723 SNPs.

The participants gave their written informed consent to participate in this study. They all got personal verbal information prior their signed consent, which was approved for this study by the Regional Research Ethics Committee. All samples were anonymized. The research was conducted according to the principles expressed in the Declaration of Helsinki.

**FIGURE 1:**
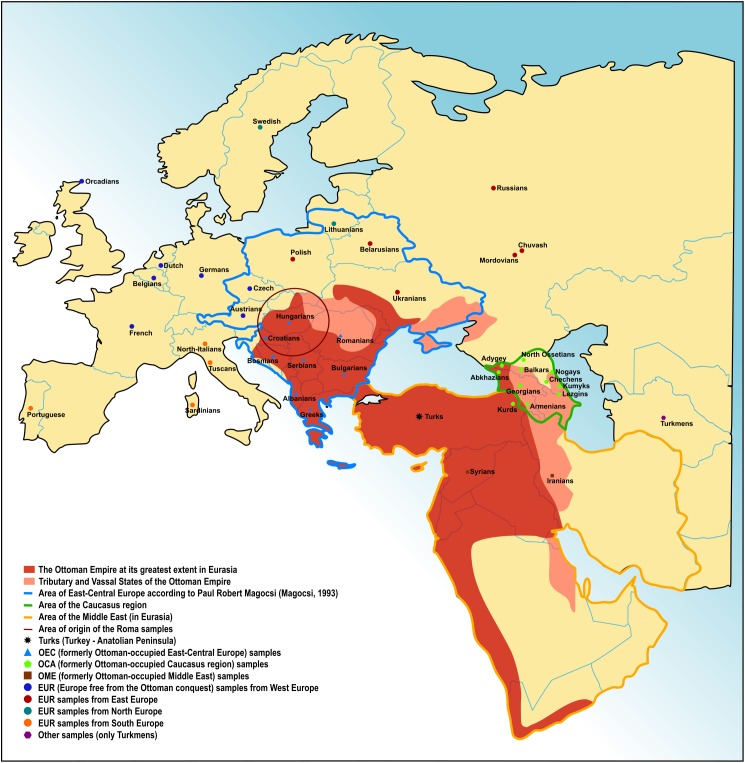
The Ottoman Empire in Eurasia. Territory of the Ottoman Empire at its greatest extent, tributary and vassal states of the Empire. Investigated regions and ethnic groups are also indicated.

The 19 Turkish samples, featuring 555,736 SNPs, were obtained from the free public repository of the Estonian Biocentre (Behar et al., 2010). Authorized access requiring, upon request available and freely available datasets were also considered in this study, of which details are described in Supplementary Table 1 (Nelson et al., 2008; Reich et al., 2009; Behar et al., 2010; Yunusbayev et al., 2012). Using these additional datasets, we created several fundamental groups for the analyses, each representing a region of Eurasia. Formerly Ottoman-occupied East-Central European (OEC) populations consisted of Albans, Bosnians, Bulgarians, Croatians, Greeks, Romanians, and Serbians (*n* = 98). We considered the definition of Paul Robert Magocsi in order to refer to the East-Central European region (Figure 1; Magocsi, 1993). Formerly Ottoman occupied populations from the Caucasus region (OCA) included Abkhazians, Adygey, Armenians, Balkars, Chechens, Georgians, Kumyks, Kurds, Lezgins, Nogays, and North Ossetia (*n* = 181). Populations from countries on the Middle East formerly under Ottoman occupation (OME) were Iranians and Syrians (*n* = 36). We created also a group for other European populations originating from all over Europe (EUR) excluding the OEC populations. This group consisted of Austrian, Basque, Belgian, Belarusian, Chuvash, Czech, Dutch, French, German, Lithuanian, Mordovian, North Italian, Orcadian, Polish, Portuguese, Russian, Sardinian, Swedish, Tuscan, and Ukrainian populations (*n* = 524). Investigated regions and populations are also shown on Figure 1. Tests using single populations from a region were also considered in some cases. Turkmens (*n* = 15) represented a Turkic Central Asian population untouched by the Ottoman conquest. Punjabi data (*n* = 221) were applied in some tests as the Indian source of Roma ancestry, Onge (*n* = 9) represented the South Asians in certain tests. Han Chinese (*n* = 44) were also applied in certain tests in order to represent Eastern Asia as an outgroup relative to the populations of the investigated Eurasian area.

### Population Structure Analysis and F_st_ Calculations

To study the relationship of the investigated populations, we implemented two different methods. The software SMARTPCA from EIGENSOFT 6.01 Software package was applied to perform principal component analysis (PCA) on our data and to compute pairwise average allele frequency differentiation (F_st_) values (Patterson et al., 2006). Clustering analysis was also performed using ADMIXTURE 1.22, which software implements a maximum likelihood estimation method to infer population ancestry distribution in a perspective of a number of hypothetical ancestral groups (Alexander et al., 2009).

For population structure and ancestry analysis, we created a merged dataset containing Turks, Roma and OEC, EUR, OCA, OME, Turkmens, Punjabi, and Han Chinese populations (*n* = 1519). As background linkage disequilibrium (background LD) can affect both PCA and ADMIXTURE analyses, marker set was thinned using PLINK v1.07 (Purcell et al., 2007). The LD-based pairwise pruning method implemented here excludes SNPs which are in strong LD. We set the pairwise genotypic correlation variable *r*^2^ to 0.5. The size of the sliding window was 50 SNPs with a sliding of 5 SNPs at a time. The thinned datasets contained 67,335 SNPs.

### Formal Tests of Admixture

To test if East-Central Europeans and Roma have Turkish ancestry, we used the algorithm implementing 4-Population Test included in the ADMIXTOOLS 4.1 Software Package (Patterson et al., 2012). The algorithm applies a 4-population test method, implemented here as D-statistics, for formal test of admixture.

For revealing admixture between East-Central Europeans and Turks, we created a dataset containing Turkish, OEC and EUR samples (*n* = 803 and 110,782 SNPs). We applied the 4-population test statistics of the tree [(OEC, Russians) (Chuvash, Turks)] to test whether East-Central Europeans and Turks are admixed.

In order to reveal admixture between Roma and Turks, we merged the data of Roma, Turks, OCA, OME, Turkmens, and Onge (*n* = 418 and 123,827 SNPs). For testing admixture between Roma and Turks, we computed the D-statistics of the unrooted phylogenetic tree [(Roma, Onge) (Turkmens, Turks)].

### Estimating Genome-Wide Ancestry Proportion of Roma

In order to test the proportion of genome-wide Turkish ancestry in Roma, we used an F_4_ ratio estimation algorithm, which is also part of the ADMIXTOOLS package. We considered the Punjabi as an ancestral population according to recent findings, which points out that Roma people originate from the Northwestern part of India (Moorjani et al., 2013; Melegh et al., 2017).

For calculating the genome-wide ancestry proportion of Turks in Roma compared to Punjabi, we used a merged dataset of Roma, Turkish, OCA, Turkmen, Punjabi, and Han Chinese samples (*n* = 638 and 124 712 SNPs).

We computed the ratio of F_4_ (Turkmens_i_, Han Chinese_i_; Roma_i_, Punjabis_i_)/(Turkmens_i_, Han Chinese_i_; Turks_i_, Punjabis_i_) and standard errors were computed using block jackknife with block size of 5 centimorgan (cM).

### Estimating Shared Identity-by-Descent (IBD) Segments

To reveal the relationship of East-Central Europeans and Roma with Turks and with other formerly Ottoman-occupied populations, and to confirm also our findings regarding the admixture events with Turks, we applied IBD segment detection using the Refined IBD method of Beagle 4.1. We created a dataset containing OEC, Roma, Turkish samples and EUR, OCA and OME groups (*n* = 1254 and 108,767 SNPs). Major alleles were set as the A1 allele with PLINK v1.07 and the dataset in binary PLINK format was converted to Variant Call Format 4.1 with the help of the PLINK/SEQ v0.10 package (Purcell, 2014). Minimum IBD segment length was set to 3 cM, the IBD trim parameter setting was 10, and an IBD scale parameter was applied according to the recommended $\sqrt{n/100}$ setting (Browning and Browning, 2013). The recommended setting applies if the data contain more than 400 individuals. Otherwise, we used an IBD scale value of 2 (Browning and Browning, 2013). We left all other parameters on its default setting.

We used the output of Beagle to compute an average pairwise IBD sharing between populations I and J.

$$\mathrm{Average pairwise IBD sharing}=\frac{\sum_{\text{i}=1}^{\text{n}}\sum_{\text{j}=1}^{\text{m}}{\text{IBD}}_{\text{ij}}}{\text{n}\times \text{m}}$$

where IBD_ij_ is the length of IBD segment shared between individuals i and j and n, m are the number of individuals in population I and J (Atzmon et al., 2010).

### Estimating Admixture Dates

In order to estimate the date of admixture of East-Central Europeans and Roma with Turks, and therefore confirm that admixture with Turks could occur between these populations during the Ottoman occupation of East-Central Europe, we applied the ALDER algorithm (Loh et al., 2013).

A merged dataset was created considering Turkish, OEC and EUR samples (*n* = 803 and 110,782 SNPs) for estimating the admixture date of OEC populations with Turks. Weighted LD decay curve fitting was applied on the setup, in which the reference populations were Portuguese and Turks, and the Hungarian samples served as the target population. Portuguese represented Europeans, which were not affected by the Ottomans or any Turkic related groups, and Hungary represented the formerly Ottoman occupied East-Central European area.

For an admixture date estimate between Roma and Turks, we created a merged dataset consisting of Onge, Turkish and Roma population samples with an SNP number of 83,459 and *n* = 88. Roma individuals were pruned according to PCA in order to obtain a most consistent ancestry for both populations. We reduced the Roma data to 30 selected individuals. We used Onge and Turks as reference populations. Onge represents the Indian ancestry of Roma people appropriately because they do not have recent West Eurasian ancestry.

Data filtering and curve fitting options were set to default values in both of the tests, and we applied the fast Fourier transform algorithm.

## Results

### Population Structure Analysis of Investigated Populations

We applied PCA using SMARTPCA and the clustering software ADMIXTURE to reveal the relationship of East-Central Europeans, Roma and Turks to each other and to other Eurasian populations. We merged the Turkish and Roma population samples into a massive dataset containing also the OEC, OCA, OME, and EUR groups. We included also Turkmen, Punjabi and Han Chinese samples in this dataset. Turkmens are Turkic people in Central Asia next to Iran, lying outside of the territory of the former Ottoman Empire at its maximum extent. Punjabi, a Northwest Indian ethnic group, represents the population of the source area of Roma. It allows us to better understand the place of Romani people in the Eurasian supercontinent. Han Chinese was used to represent East Asian populations in the Eurasian perspective.

**FIGURE 2:**
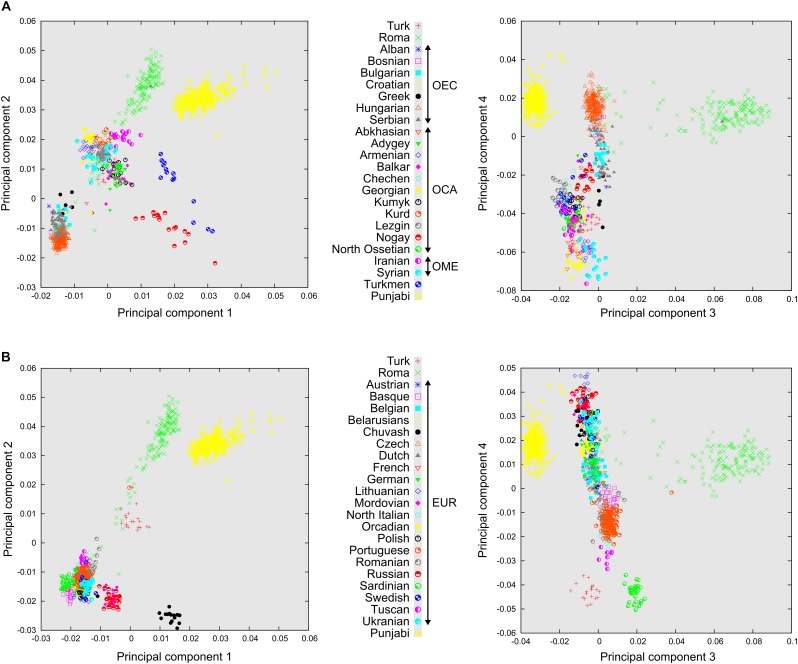
Relationship of Turks and Roma to distinct Eurasian Populations according to PCA. PCA results plotted on four principal components. Each symbol represents one individual. **(A)** Relationship of Turks to formerly Ottoman occupied populations in East-Central European populations (OEC), Caucasus region populations (OCA), Middle Eastern populations (OME), and also Romani people. **(B)** Relationship of Turks and European populations not affected by the Ottoman conquest (EUR). Note that these PCA graphs are the results of the same PCA analysis.

Principal component analysis results, plotted on the first four principal components, show the three main groupings of the analyzed populations, which are Europeans, populations of the Caucasus region together with Middle East populations and the South Asian ethnic group Punjabi. Roma people cluster mostly between the Caucasus populations and Punjabi. There are also several more admixed Roma individuals scattered throughout a straight line of which two ends are defined by Europeans and South Asians (Figure 2A). We can also observe that most of the OEC populations cluster closer to the populations of the Caucasus region. Turkmens and Nogays show significant East Asian ancestry, since they cluster in the direction of Han Chinese samples, which constitute a separate group far from the investigated populations, and are therefore included only on ADMIXTURE graphs. European populations that did not fall under Ottoman rule (or more obviously, are located significantly farther from Turkey) cluster farther from Turks (Figure 2B). Sardinians and Basques cluster separately from other European populations, showing some degree of genetic isolation. The Turkic Chuvash group living in Russia, similarly to Turkmens, also have notable East Asian ancestry. In the PCA analysis, eigenvalues of the eigenvectors (principal components) 1 and 2 were 26.56 and 10.54, eigenvectors 3 and 4 were 6.49 and 4.75, respectively. Eigenvalue of 10 principal components were higher than 2.00, and eigenvalue of 652 principal components were higher than 1.00.

**FIGURE 3:**
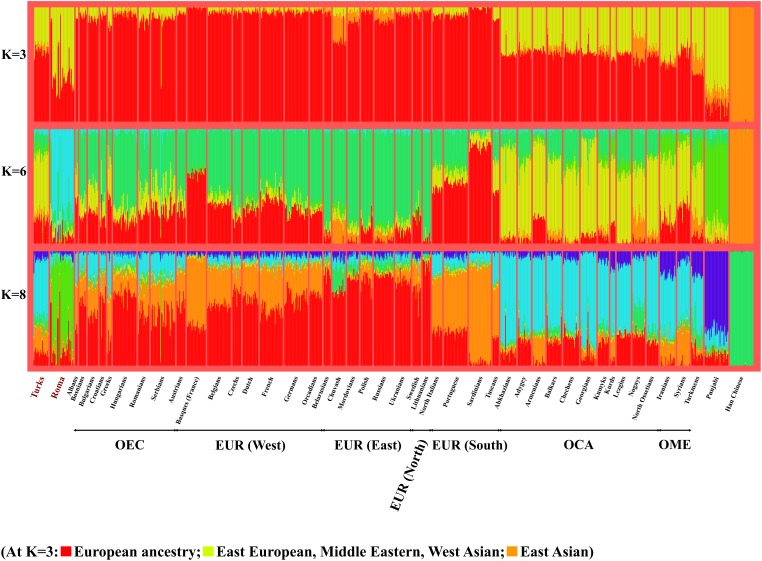
Relationship of Turks and Roma to various Eurasian populations according to ADMIXTURE analysis. ADMIXTURE analysis results with *K* = 3, 6, and 8 hypothetical ancestral groups. The number of displayed individuals was limited to 30 at each group. ADMIXTURE results with *K*-values 3–10 can be found in the Supplementary Figure 1.

The ADMIXTURE analysis produced similar results and visualizes the data similarly to PCA in a stacked column chart style. At *K* = 3, which showed the highest decrease of cross-validation error, we can observe the population relationships that PCA showed (Figure 3). Red color indicates European ancestry, yellow likely indicates an East European-West Asian ancestry and orange represents an East Asian hypothetical ancestral group. Populations living in the Caucasus region and in the Middle East are well-separated from European populations, since Caucasus populations have on average a 32.6% proportion from the East European-West Asian ancestry, while Europeans have only 3.2 and 8.4% in case of EUR and OEC groups. These slight differences in the proportion of East European-West Asian ancestry could also indicate the different history of East-Central European and other European populations, likely showing the more significant influence of the Middle East in East-Central Europe. Turks belong the Middle East area, therefore they have similar ancestry proportion from the hypothetical groups than of populations living in the Caucasus and in the Middle East. The proportion of Turks from the East European-West Asian ancestry is 33.2%. Roma samples, which show significant admixture with non-Roma Europeans, appear also on the ADMIXTURE graphs. Supplementary Figure 1 shows the ADMIXTURE analysis results applying *K* = 3 to 10 hypothetical ancestral groups.

### Pairwise Average Allele Frequency Differentiation Estimations

We computed the genome-wide pairwise average allele frequency differentiation values (F_st_) between the investigated populations. Examining F_st_ values between Turks and OEC, EUR populations show that OEC have lower F_st_ with Turks than the F_st_ between Turks and EUR populations (Table 1). Turks have the lowest F_st_ with OCA and OME groups, as expected. Roma have lower F_st_ with Turks than OCA and OME populations and Turkmens. Sardinians were represented separately from the EUR group on the table. Sardinians are a South European group, which live isolated from the demographic events of Europe, as population structure analysis showed. Therefore, they are also less affected by the effects of Ottoman occupation of East-Central Europe.

**Table 1:** Population relationships based on pairwise average allele frequency differentiation (F_st_) estimations.

	Turks	Roma	OEC	OCA	OME	EUR	Sardinians
Turks							
Roma	0.011						
OEC	0.006	0.014					
OCA	0.004	0.014	0.009				
OME	0.003	0.014	0.008	0.006			
EUR	0.009	0.017	0.005	0.013	0.016		
Sardinians	0.013	0.023	0.009	0.019	0.016	0.012	
Turkmens	0.013	0.021	0.017	0.015	0.014	0.022	0.02

### Testing for Admixture

A formal test of admixture, the 4-population test, was applied to find evidence that East-Central Europeans and Roma have admixed with Turks (Patterson et al., 2012).

In case of East-Central Europeans, accordingly to the PCA results, we used Russian individuals from European populations that were never affected by the Ottoman rule. The Turkic people Chuvash from East Europe was also considered in the test to try to rule out other possible Turkic ancestry. We tested if the phylogenetic tree, on which East-Central Europeans to East Europeans and Turks to Turkic groups are closely related. The 4-population test showed a violation of the expected phylogenetic tree, confirming that East-Central-Europeans are admixed with Turks.

In order to examine whether Roma and Turks are admixed, we considered Onge samples, which group is an accurate surrogate of the South Asian ancestry of Roma. Since recent admixture between Onge and West Eurasians did not occur, European ancestry components cannot influence our test (Moorjani et al., 2013). Besides Onge, we used Turkmens as a Turkic group closely related to Turks. The 4-population test showed also a significant violation in this setup and confirmed that Roma are also admixed with Turks (Supplementary Table 2).

We applied F_4_ ratio estimation to measure the proportion of Turkish ancestry in the Roma (Patterson et al., 2012). In our setup, we calculated the Turkish ancestry proportion of Roma using Punjabi as second source population of the admixture, and using Turkmens as a sister group of Turks, which are the least admixed with Roma, according to our analyses. Han Chinese were considered as outgroup in this test. F_4_ ratio estimation shows that Roma have 66.5 ± 2.7% Turkic related ancestry compared to their Northwestern Indian origin (*z* = 24.25, standard error = 0.027) (Supplementary Table 3).

### Confirming Admixture With Average Pairwise Identity-by-Descent Segment Sharing Estimation

**FIGURE 4:**
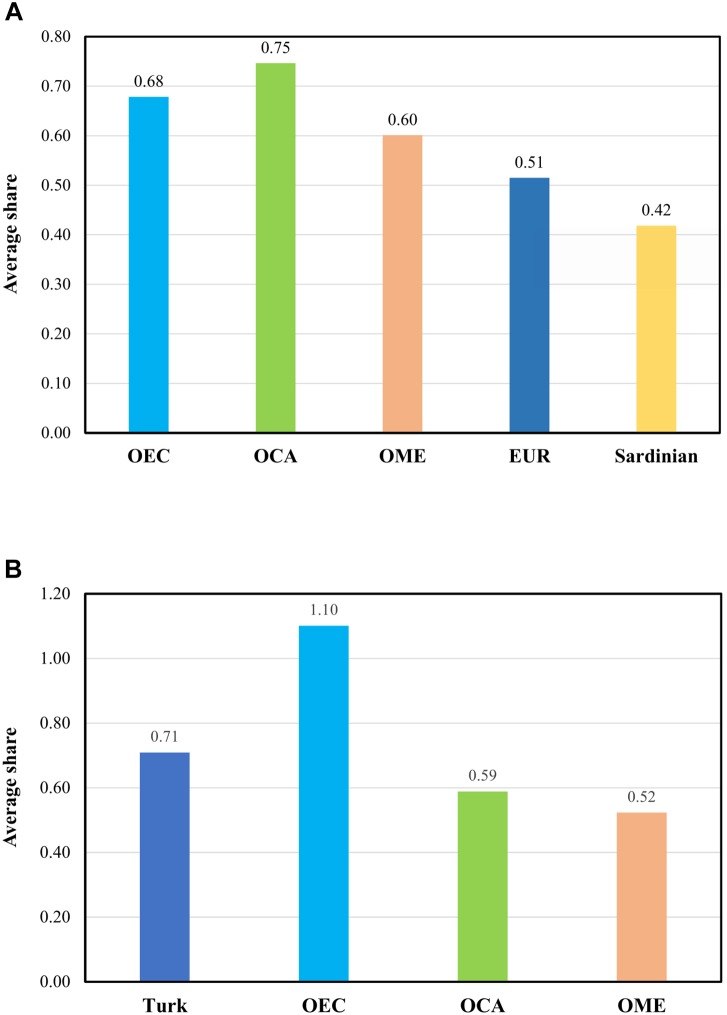
Population relationships based on average IBD sharing estimations. We computed the genome-wide pairwise average shared IBD length between certain groups. OEC – Formerly Ottoman-occupied East Central Europe, OCA – Formerly Ottoman-occupied Caucasus region, OME – Formerly Ottoman-occupied Middle East, EUR – Europe free from Ottoman conquest. **(A)** Average shared IBD length between Turks and the formerly Ottoman occupied groups, Europeans not affected by the Ottoman Empire displayed separately the Sardinians. **(B)** Average shared IBD length between Roma and Turks and formerly Ottoman occupied populations. A more detailed table of the average IBD sharing data are available in Supplementary Table 4.

To detail further the relationship of East-Central Europeans and Roma to Turks, and assess the Turkish ancestry in East-Central Europeans and Roma, we estimated the average pairwise Identity-by-descent segment sharing between certain populations. We created a merged dataset of the Turks, Roma and groups living in the formerly Ottoman-ruled regions, as well as EUR populations. OEC populations have an average pairwise IBD share of 0.68 with Turks. This means that East-Central Europeans have similarly higher average IBD sharing with Turks than the populations of formerly Ottoman-occupied Caucasus and Middle Eastern regions, which was 0.75 and 0.60, respectively. The average sharing between other Europeans and Turks and between Sardinians and Turks were 0.51 and 0.42, respectively. These results suggest that Ottoman rule in East-Central Europe has a detectable impact on its populations (Figure 4A).

In order to further investigate Roma admixture with Turks during the Ottoman rule, we compared the average IBD sharing with Turks and OCA. The migration route of Roma included Turkey and the neighboring Caucasus region, therefore the rate of admixture with Turks and populations from the Caucasus should be close to each other if an admixture between Roma and Turks did not occur during the Ottoman occupation. However, IBD analysis shows a higher average IBD sharing with Turks (0.71) than Caucasus populations (0.60), likely confirming the assumption that admixture between Roma and Turks occurred during the Ottoman occupation (Figure 4B).

### Estimating Admixture Dates

**FIGURE 5:**
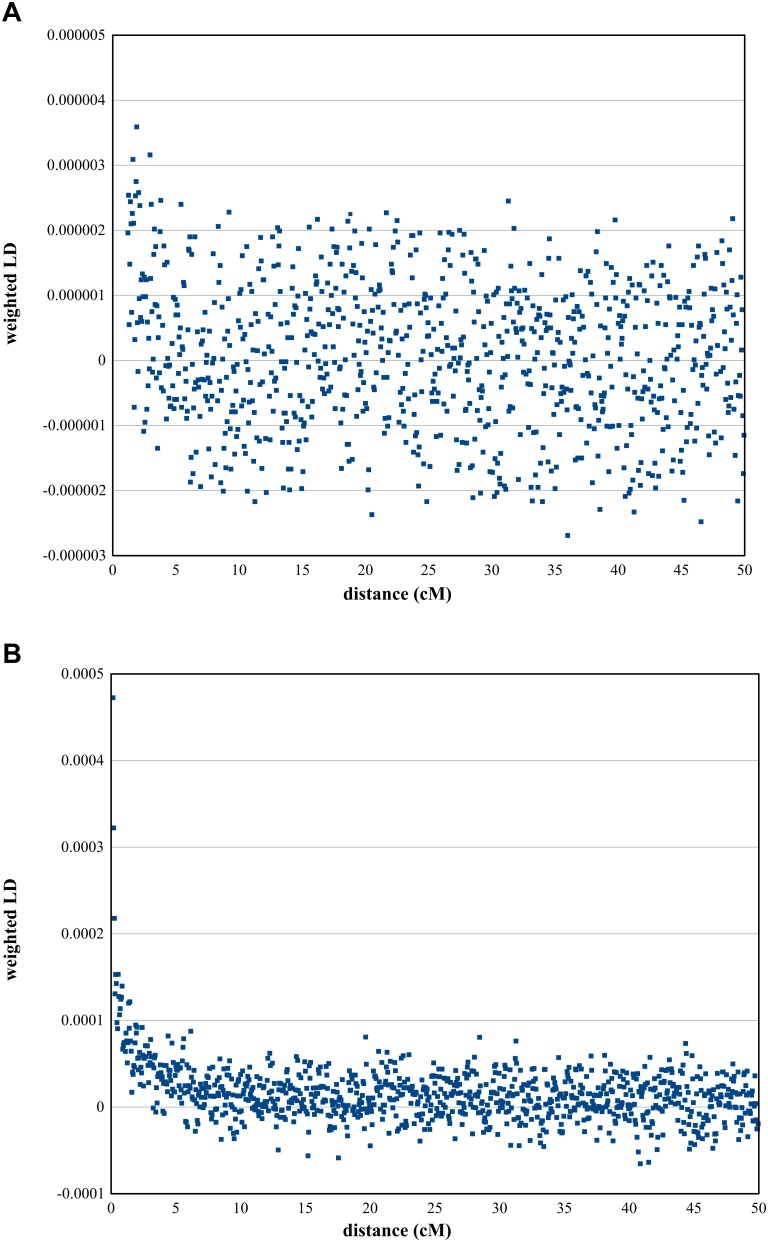
ALDER analysis results. Weighted LD curves calculated by the ALDER algorithm. **(A)** Weighted LD curve of Hungarians representing OEC and the two reference populations Turks and Portuguese. **(B)** Result of ALDER analysis using Roma as target group and two reference populations, Turks and Onge.

In order to further investigate Roma admixture with Turks, we used ALDER to estimate admixture dates. Preliminary ALDER analyses applying multiple reference populations showed that Portuguese samples are appropriate surrogates for representing a population not affected by the Ottoman era and Hungarians are an appropriate surrogate of populations from the formerly Ottoman occupied East-Central European region. According to the results of ALDER, admixture between East-Central Europeans occurred 18.09 ± 5.33 generations ago (*z* = 2.35; *p* = 0.019) (Figure 5A). If one generation equals 29 years (Fenner, 2005), admixture of East-Central Europeans and Turks dates back to 370–679 years ago, which closely approaches the time frame when Ottomans occupied the East-Central European region.

Admixture between Roma and Turks occurred 30.56 ± 3.77 generations or approximately 777–996 years ago (*z* = 4.1, *p* = 4.1 × 10^−5^) (Figure 5B). This is consistent with historical data regarding their appearance in the Pannonian basin, and can also indicate that Roma could have also admixed with Turks during their migration in Anatolia.

## Discussion

In order to better understand the relationship of Roma, East-Central European populations and Turks to each other and in a Eurasian perspective, we applied the population structure and ancestry analysis methods PCA and ADMIXTURE. These methods helped to place the investigated populations in a Eurasian context, which showed that East-Central Europeans and Turks belong to two well-separated groups. Roma, due to their nomadic nature, scattered severely between Europe and South Asia. However, most Roma individuals clustered somewhat more tightly to each other, scattering between South Asia and the populations of the Caucasus, Middle East and Central Asia. PCA and ADMIXTURE results reflected the actual geographical positions of East-Central Europeans and Turks. Ancestry analysis and F_st_ calculations showed that East-Central Europeans have significant Turkish ancestry, even compared to other formerly Ottoman-occupied East-Central European populations. These analyses also revealed that Roma might have remarkable Turkish ancestry compared to the populations of neighboring regions of Turkey, which also fall on the migration route of the ancestors of recent Roma.

Based on the results of PCA and ADMIXTURE, we investigated the proposed admixture events with formal test of admixture between Roma, East-Central Europeans and Turks, which could provide evidence of gene flow between these populations. In case of East-Central Europeans, we used East European Slavic and Turkic population samples to find evidences of admixture with Turks. The 4-population test results showed that gene flow between East Europeans and Turks occurred. This result suggests that the test revealed Turkish ancestry from the Ottoman occupation of East-Central European territories. Estimating the average IBD sharing of East-Central Europeans with Turks showed that East-Central European populations have higher average IBD sharing with Turkic people than other formerly Ottoman-occupied populations. The 4-population test showed also that Roma and Turks are admixed, and as we did in case of East-Central Europeans, we confirmed with average IBD sharing estimation that this admixture could originate from the former Ottoman rule of East-Central Europe, since Turkish ancestry in Roma shows a higher degree than the ancestry of populations living adjacent regions of Turkey.

To assess the significance of average IBD sharing difference values between investigated populations or groups, we also calculated the average IBD sharing of Turks with Sardinians, which group lives separately from the continent on the Sardinia Island, therefore they are largely isolated from European demographic events. This investigation suggested that average IBD sharing differences of investigated populations with Turks are significant.

We estimated also the date of the admixtures in order to further investigate and provide evidence for the proposed admixture of East-Central Europeans and Roma with Turks during the Ottoman occupation. In case of East-Central Europeans, the obtained admixture date interval corresponds to the time interval when Ottomans were present in East-Central Europe. Ottoman presence in the region solidified approximately with the Battle of Kosovo (1389), and fall of Ottoman rule in East-Central Europe is associated with the successful campaign led by the Habsburgs, which resulted in the Treaty of Karlowitz signed in 1699 (Elsie, 2004; Abou-El-Haj, 1967; Abou-el-Haj, 1969). This analysis strengthened our proposal that Turkish ancestry in East-Central Europeans can originate from the Ottoman presence in Europe, and ancestry from Turkic people in Roma could be derived also from the times of the Ottoman occupied Europe.

We confirmed that the expansion of the Ottoman Empire into East-Central Europe left its mark on local populations, contributing significantly to their Turkic ancestry. Using genome-wide autosomal SNP array data, we were able to find a similar geographical pattern in the genetic makeup of European populations, as studies based on Y haplogroups found. Population structure showed that Western and Eastern European populations are well-separated from each other. Ancestry analysis, testing for admixture and IBD segment analyses further separated OEC groups from the rest of Europe, showing that ancestry derived from the Middle Eastern area is also observable in autosomal data. As the Middle Eastern region derived Y haplogroups E3b and J were observed in high extent in East-Central Europeans (Cruciani et al., 2004; Semino et al., 2004; Battaglia et al., 2009), we found that Middle Eastern ancestry shows also the highest extent in OEC groups in case of genome-wide autosomal marker data. We revealed also that Romani people could have acquired Turkish ancestry not only during their migration to Europe, but also at the time of Ottoman presence in Europe, when they were already the largest ethnic minority of the area.

## Ethics Statement

This study was carried out in accordance with the recommendations of the Hungarian Human Genetic Law (act XXI/2008) with written informed consent from all subjects. All subjects gave written informed consent in accordance with the Declaration of Helsinki. The protocol was approved by the Research Ethics Committee in Pécs (REKEB).

## Author Contributions

ZB, BIM, and KS conceived and designed the investigations. ZB and BIM evaluated and interpreted the results. KH, KS, and BM contributed in the data collection and genotyping. ZB, BM, AM, and MK cowrote the manuscript. BM, AM, and MK revised the initial draft of the manuscript. All authors were involved in this work.

## Conflict of Interest Statement

The authors declare that the research was conducted in the absence of any commercial or financial relationships that could be construed as a potential conflict of interest.
