# Supplementary material for: Revealing the Genetic Impact of the Ottoman Occupation on Ethnic Groups of East-Central Europe and on the Roma Population of the Area
Source: Front Genet. 2019 Jun 13;10:558. doi: 10.3389/fgene.2019.00558 (PMC6585392; doi:10.3389/fgene.2019.00558)
Supplement: Supplementary file 1 [file Data_Sheet_1.PDF]

Supplementary Table 1. Summary of the applied datasets and groups created for the analyses

Dataset name	Dataset source	Dataset availability	Group/population count	Number of samples used	Groups used	Study roles	Comment
**Roma data #1**	Department of Forensic Molecular Biology, Erasmus MC University Medical Center Rotterdam, Rotterdam, The Netherlands	The Roma genome-wide marker data from the Mendizabal et al. 2012 paper are fully available upon reasonable request at the corresponding author of the original paper at Erasmus MC University Medical Center Rotterdam without any special author access requirements (contact: m.kayser@erasmusmc.nl).	1	**132**	**1**<br>Roma	Roma population	-
**Roma data #2**	Department of Medical Genetics, University of Pécs, Pécs, Hungary	The access of the Roma genome-wide marker data used in the Moorjani et al. 2013 paper are available upon a reasonable research proposal. Requesting the data can be initiated at the contact of the corresponding author (melegh.bela@pte.hu).	1	**27**	1<br>Roma	Roma population	-
**Hungarian data**	Department of Medical Genetics, University of Pécs, Pécs, Hungary	The access of the Hungarian genome-wide marker data are available upon a reasonable research proposal. Requesting the data can be initiated at the contact of the corresponding author (melegh.bela@pte.hu).	1	**238**	**1**<br>Hungarian	OEC	-
**Caucasus Paper Data**	*Yunusbayev et. Al. 2012* (can be found in the references)	http://evolbio.ut.ee/caucasus/	13	**189**	**12**<br>**Europe:** Bulgarian, Mordovian, Ukranian<br>**Caucasus:** Abkhasian, Armenian, Balkar, Bulgarian, Chechen, Kumyk, Kurd, Nogay, North Ossetian<br>**Middle East (or Central Asia):** Turkmen	Source of EUR, OCA, OEC and Turkmens	-
**Jew Data**	*Behar et. Al. 2010* (can be found in the references)	http://evolbio.ut.ee/jew/	39	**129**	**8**<br>**Turks**<br>**Europe**: Belorusian, Chuvash, Lithuanian<br>**Caucasus:** Georgian, Lezgin<br>**Middle East:** Iranian, Syrian	Source of Turkish population, EUR, OCA, OME	-
**HGDP data**	Stanford University	http://www.hagsc.org/hgdp/files.html	52	**204**	**9**<br>**Europe:** Basque, French, North Italian, Orcadian, Russian, Sardinian, Tuscan<br>**Caucasus:** Adygey<br>**East Asia:** Han Chinese	Source of EUR, OCA and Han Chinese	-
**POPRES data**	NCBI dbGaP	The dataset are not publicly available due to the limitation of authorized access requirements, but authorization process can be initiated at the repository of NCBI (see Acknowledgements)	57	**616**	**15**<br>**Europeans:** Alban, Austrian, Belgian, Bosnian, Croatian, Czech, Dutch, German, Greek, Polish, Portuguese, Romanian, Swedish, Serbian<br>**Indians:** Punjabi	Source of EUR, OEC and Punjabi	-
**Indian data**	David Reich, Harvard Medical School, Department of Genetics, Boston, USA	The Indian genome-wide marker data from the Reich et al. 2009 paper are fully available upon request without any special author access requirements at the corresponding author of the original paper at Department of Genetics, Harvard Medical School, Boston, Massachusetts, United States of America (contact: reich@genetics.med.harvard.edu).	23	**9**	**1**<br>Indian: Onge	Source of Onge population	applied only in 4 Population Tests
