# Supplementary material for: Revealing the Genetic Impact of the Ottoman Occupation on Ethnic Groups of East-Central Europe and on the Roma Population of the Area
Source: Front Genet. 2019 Jun 13;10:558. doi: 10.3389/fgene.2019.00558 (PMC6585392; doi:10.3389/fgene.2019.00558)
Supplement: Supplementary file 2 [file Data_Sheet_2.PDF]

Supplementary Table 2. D-statistics calculations of proposed OEC, Turk and Roma admixture events

population W	*population X*	*population Y*	*population Z*	*D-statistics*	*Z-score*
OEC	Russian	Chuvash	Turk	-0.012	-20.184
Russian	OEC	Chuvash	Turk	0.012	20.184
Roma	Onge	Turkmen	Turk	-0.0261	-21.855
Onge	Roma	Turkmen	Turk	0.0261	21.855
