# Supplementary material for: Revealing the Genetic Impact of the Ottoman Occupation on Ethnic Groups of East-Central Europe and on the Roma Population of the Area
Source: Front Genet. 2019 Jun 13;10:558. doi: 10.3389/fgene.2019.00558 (PMC6585392; doi:10.3389/fgene.2019.00558)
Supplement: Supplementary file 3 [file Data_Sheet_3.PDF]

Supplementary Table 3. F_4 statistics results. Calculations of Turkish ancestry proportion in Romani people compared to Northwest Indian ancestry.

group A	*Outgroup*	*group X*	*group C*	*group A*	*Outgroup*	*group B*	*group C*	*Alpha*	*Standard Error*	*Z-score*
**Turkmens**	**Han Chinese**	**Roma**	**Punjabi**	**Turkmens**	**Han Chinese**	**Turks**	**Punjabi**	**0.665734**	**0.027452**	**24.251**
Abkhasians	Han Chinese	Roma	Punjabi	Abkhasians	Han Chinese	Turks	Punjabi	0.668952	0.020649	32.396
Adygey	Han Chinese	Roma	Punjabi	Adygey	Han Chinese	Turks	Punjabi	0.683021	0.021995	31.054
Armenians	Han Chinese	Roma	Punjabi	Armenians	Han Chinese	Turks	Punjabi	0.680023	0.019339	35.163
Balkars	Han Chinese	Roma	Punjabi	Balkars	Han Chinese	Turks	Punjabi	0.688284	0.021463	32.068
Chechens	Han Chinese	Roma	Punjabi	Chechens	Han Chinese	Turks	Punjabi	0.689869	0.022889	30.14
Georgians	Han Chinese	Roma	Punjabi	Georgians	Han Chinese	Turks	Punjabi	0.675669	0.020284	33.311
Kumyks	Han Chinese	Roma	Punjabi	Kumyks	Han Chinese	Turks	Punjabi	0.689577	0.023151	29.787
Kurds	Han Chinese	Roma	Punjabi	Kurds	Han Chinese	Turks	Punjabi	0.670743	0.024171	27.75
Lezgins	Han Chinese	Roma	Punjabi	Lezgins	Han Chinese	Turks	Punjabi	0.667171	0.023277	28.663
Nogays	Han Chinese	Roma	Punjabi	Nogays	Han Chinese	Turks	Punjabi	0.710511	0.022994	30.9
North Ossetians	Han Chinese	Roma	Punjabi	North Ossetians	Han Chinese	Turks	Punjabi	0.672770	0.021434	31.388
