# Supplementary material for: Revealing the Genetic Impact of the Ottoman Occupation on Ethnic Groups of East-Central Europe and on the Roma Population of the Area
Source: Front Genet. 2019 Jun 13;10:558. doi: 10.3389/fgene.2019.00558 (PMC6585392; doi:10.3389/fgene.2019.00558)
Supplement: Supplementary file 4 [file Data_Sheet_4.PDF]

Supplementary Table 4. Average pairwise IBD share between populations

Group	Population	Average IBD share with Turks	Average IBD share with Roma	Average IBD share of groups with Turks	Average IBD share of groups with Roma
	Turk	-	0.71	-	-
	Roma	0.71	-	-	-
OEC	Bosnian	0.53	1.06	0.68	1.10
	Bulgarian	0.63	0.84		
	Croatian	0.74	0.87		
	Greek	0.81	0.52		
	Hungarian	0.69	2.22		
OCA	Abkhasian	0.81	0.50	0.75	0.59
	Adygey	0.77	0.80		
	Armenian	0.78	0.62		
	Balkar	0.52	0.64		
	Chechen	0.84	0.64		
	Georgian	0.93	0.54		
	Kumyk	0.76	0.61		
	Kurd	0.76	0.40		
	Lezgin	0.73	0.58		
	Nogay	0.59	0.63		
	North Ossetian	0.73	0.52		
OME	Iranian	0.63	0.63	0.58	0.52
	Syrian	0.53	0.41		
EUR	Basque	0.51	0.65	0.51	0.78
	Dutch	0.49	0.75		
	Portuguese	0.54	0.97		
	Swedish	0.51	0.76		
	Sardinian*	0.42	0.52	-	-

OEC	0.68
OCA	0.75
OME	0.60
EUR	0.51
Sardinian	0.42

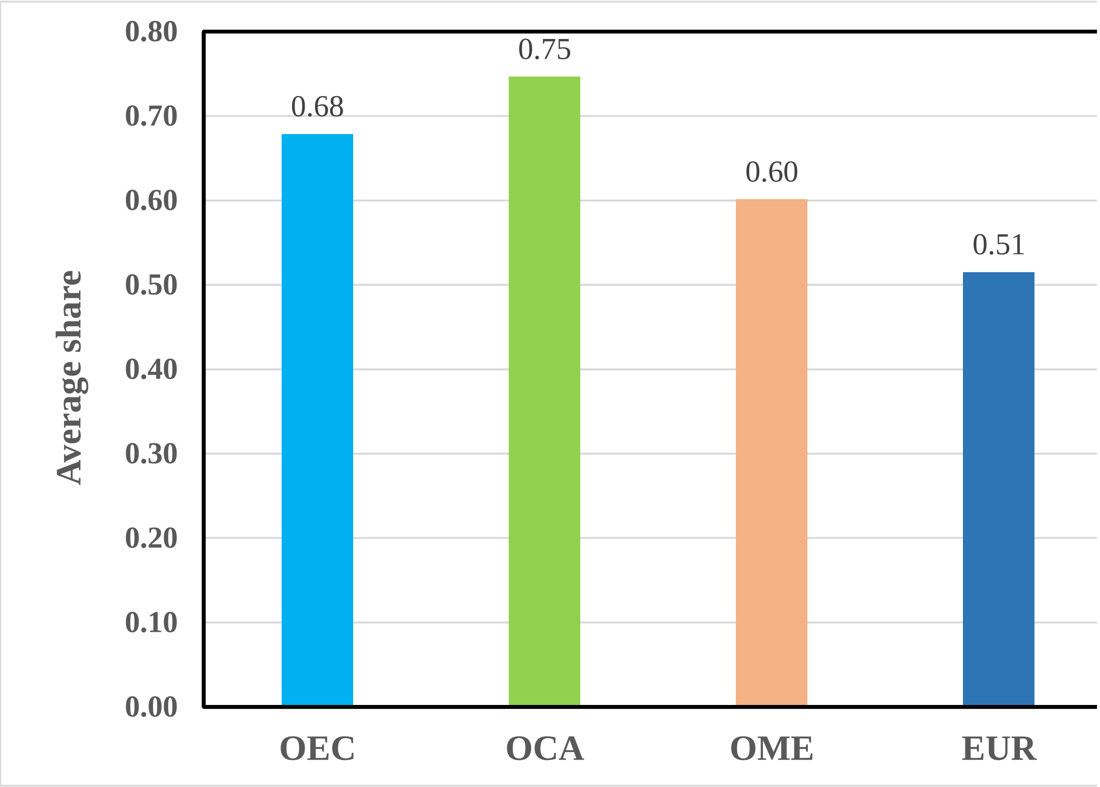

Turk	0.71
OEC	1.10
OCA	0.59
OME	0.52

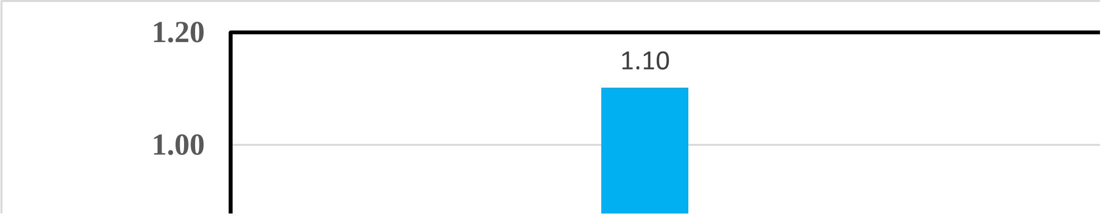

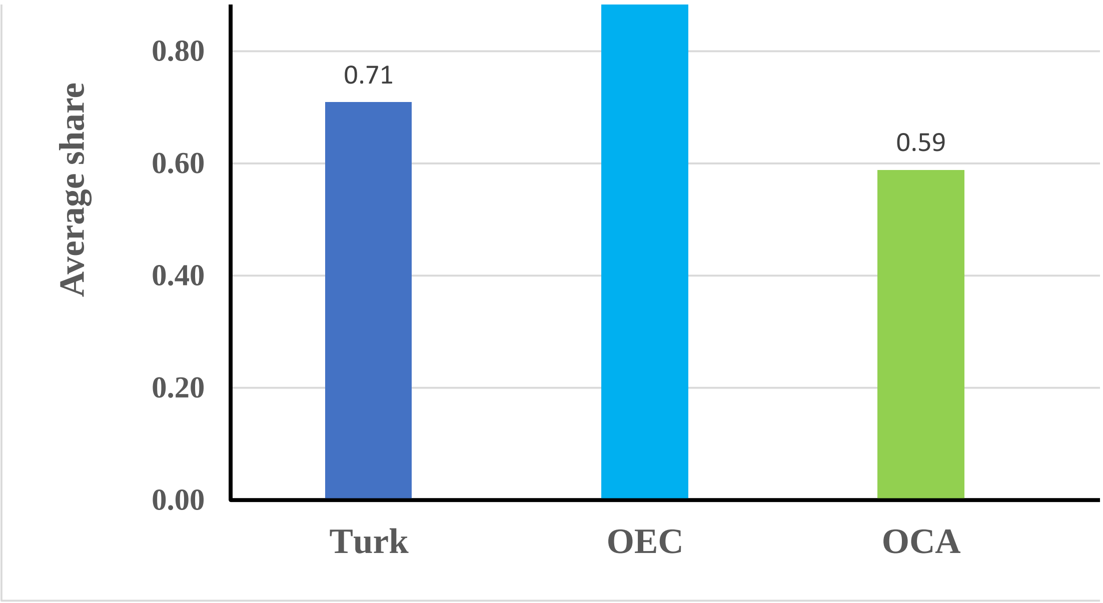
Average share
0.80
0.60
0.40
0.20
0.00
0.71
0.59
Turk
OEC
OCA

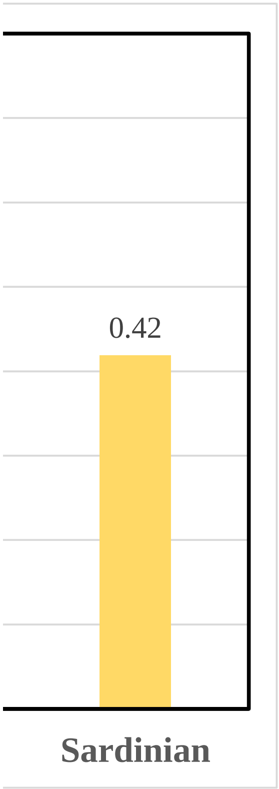
0.42
Sardinian

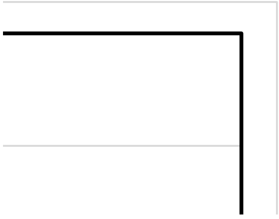

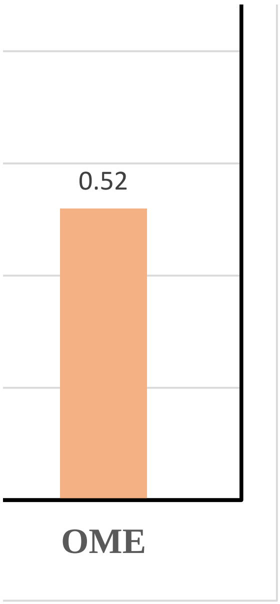
0.52
OME
